# Development and Characterization of Honey- and Essential Oil-Based Structured Systems for Skin Applications

**DOI:** 10.3390/ph19071103

**Published:** 2026-07-17

**Authors:** Corina-Bianca Ioniță-Mîndrican, Manuela Ghica, Ancuța Cătălina Fița, Eliza Oprea, Mihaela Buleandră, Irinel Adriana Badea, Cristina-Ionela Stănciulescu, Emma Adriana Ozon, Silviu-Iulian Filipiuc, Carolina Negrei

**Affiliations:** 1 Department of Toxicology, Faculty of Pharmacy, “Carol Davila” University of Medicine and Pharmacy, 6 Traian Vuia St., 020956 Bucharest, Romania; corina-bianca.ionita-mindrican@drd.umfcd.ro (C.-B.I.-M.); carolina.negrei@umfcd.ro (C.N.); 2 Department of Biostatistics, Faculty of Pharmacy, “Carol Davila” University of Medicine and Pharmacy, 6 Traian Vuia St., 020956 Bucharest, Romania; manuela.ghica@umfcd.ro; 3 Department of Pharmaceutical Technology and Biopharmacy, Faculty of Pharmacy, “Carol Davila” University of Medicine and Pharmacy, 6 Traian Vuia St., 020956 Bucharest, Romania; emma.budura@umfcd.ro; 4 Department of Botany and Microbiology, Faculty of Biology, University of Bucharest, 1-3 Portocalelor, 77206 Bucharest, Romania; 5 Department of Analytical Chemistry and Physical Chemistry, Faculty of Chemistry, University of Bucharest, 90–92 Panduri Street, 050663 Bucharest, Romania; mihaela.buleandra@g.unibuc.ro (M.B.); irinel.badea@chimie.unibuc.ro (I.A.B.); 6 Dermatology Department, Faculty of Medicine, University of Medicine and Pharmacy Craiova, 2 Petru Rares Street, 200349 Craiova, Romania; cristina.stanciulescu@umfcv.ro; 7 Advanced Research and Development Center for Experimental Medicine “Prof. Ostin C. Mungiu”—CEMEX, Grigore T. Popa University of Medicine and Pharmacy Iasi, 9-13 Mihail Kogălniceanu Street, 700454 Iasi, Romania; silviu.filipiuc@umfiasi.ro

**Keywords:** topical structured systems, honey, natural cosmetic, skin hydration, skin elasticity, skin rejuvenation, vegetable oils, essential oil

## Abstract

**Background**: The present study aimed to develop and characterize three honey- and essential oil-based structured systems intended for topical skin application. **Materials and Methods**: The semisolid systems were prepared as oil-in-water structured emulsions containing four types of honey (Manuka, Tualang, chestnut, and manna), three essential oils (palmarosa, cistus, and lavender), and five vegetable oils (pomegranate seed, aloe, centella, hemp seed, and calendula). Each formulation consisted of two honey types, one essential oil, and two vegetable oils, with final concentrations of 5% honey and 0.1–0.2% essential oil. The formulations were investigated through physicochemical, rheological, and in vivo skin evaluations. **Results**: Rheological analysis demonstrated non-Newtonian pseudoplastic behavior with shear-thinning and thixotropic characteristics, indicating that the systems are structured, semisolid, and suitable for topical application. Differences in spreadability and consistency suggested variations in the internal organization of the emulsion matrices. In vivo skin assessments were performed over four weeks using non-invasive instrumental methods. The obtained results demonstrated improvements in skin hydration, elasticity, firmness, and skin barrier function, together with reductions in transepidermal water loss and erythema. The evaluation of skin textural parameters revealed improvements in skin uniformity and a reduction in the appearance of wrinkles. Among the tested structured systems, F3 formulation exhibited the most pronounced moisturizing effect, while F1 formulation showed notable improvements in parameters associated with skin texture and wrinkle-related features. **Conclusions**: Overall, the results indicate that honey-based topical systems enriched with essential and vegetable oils represent promising multifunctional semisolid formulations for topical skin-conditioning and barrier-supportive applications. Their favorable rheological behavior, combined with beneficial effects on skin hydration, barrier function, and skin surface properties, supports their potential use in skin-conditioning and anti-aging-related formulations.

## 1. Introduction

In recent times, the cosmetics industry based on natural compounds has experienced significant growth. In ten years, from 2023 to 2033, the market is estimated to nearly double from USD 48.4 billion in 2023 to USD 79.6 billion by 2033. This upward trend is explained by the desire to develop clean beauty, which emphasizes the use of products that are free of harmful substances and environmentally sustainable. There is an observed trend of consumers seeking products that are increasingly clean and beneficial for their skin [1,2].

In dermatocosmetic formulations, honey is considered a valuable natural ingredient due to its wide availability, consumer acceptance, and well-documented benefits for skin health. It is extensively used worldwide not only as a nutritional product but also in dermatological and cosmetic applications. Honey possesses a complex chemical profile, consisting predominantly of simple sugars such as glucose and fructose, together with a range of bioactive compounds, including flavonoids, phenolic substances, vitamins, and minerals, which contribute to its functional properties [3,4,5,6]. Furthermore, honey demonstrates significant antimicrobial and antioxidant activities, supporting its suitability for topical formulations. Its application has been associated with the improvement of various skin conditions, particularly those involving inflammation, irritation, or tissue damage [1,7,8].

A survey conducted on 487 participants showed that approximately 42.9% reported the topical use of honey. Most respondents indicated occasional use, with around 27% applying it several times a year or less frequently, while smaller proportions reported monthly (8.1%) or more frequent applications. Among individuals who used honey in cosmetic applications (*n* = 209), it was primarily associated with skin hydration (approximately 23%), followed by soothing effects on skin irritation (19.1%), improvement of skin elasticity (17.7%), and anti-aging benefits (16.8%). These findings highlight the perceived multifunctional role of honey in skincare practices [7].

A study investigating the effects of multifloral honey-based creams on skin para-meters demonstrated that, following four weeks of regular application, significant im-provements in skin hydration (up to 29.7%) were observed, along with a reduction in wrinkle area (up to 21.4%) [1].

For the preparation of the formulations in this study, Manuka honey (New Zealand), Tualang honey (Malaysia), chestnut honey (Spain), and manna honey (Romania) were selected. Previous results confirmed both their authenticity and botanical characteristics. All four honey types showed a water content below 20% and appropriate diastase activity values: Manuka (17.90 ± 2.28), Tualang (10.90 ± 1.39), chestnut honey (29.40 ± 3.75), and manna honey (38.50 ± 4.90). Additionally, melissopalynological analysis highlighted the characteristic pollen spectrum of each honey type. Manuka honey showed a predominance of pollen types associated with the Myrtaceae family (73.16%), while chestnut honey was characterized by a high proportion of pollen types belonging to the Fagaceae family (94.17%). Tualang honey presented a pollen spectrum predominantly associated with the Arecaceae and Fabaceae families, whereas manna honey exhibited a diverse pollen spectrum including pollen types associated with Rosaceae, Myrtaceae, Brassicaceae, and Ericaceae [5].

Recently, skincare products have focused on the development of products with regenerative properties. Thus, essential oils are of interest due to their high content of bioactive compounds. Recent studies have shown that volatile oils and their monoterpene components can act as anti-aging agents, exerting antioxidant and anti-inflammatory effects by inhibiting the NF-κB signaling pathway. Skin integrity is protected by the inhibition of degradative enzymes, such as elastase and matrix metalloproteinases, involved in collagen breakdown [9,10,11]. For example, essential oils from the Cistus species can reduce UVB-induced cellular senescence, while also enhancing the expression of SIRT1, an anti-aging gene [9,12]. Meanwhile, Lavender oil (through linalool) is recognized for its anti-inflammatory potential [9].

Other components used for the development of formulators were allantoin, bisabolol, and hyaluronic acid. It is known that allantoin can stimulate collagen synthesis [13], while bisabolol can reduce inflammation [14]. It is also demonstrated that hyaluronic acid has moisturizing, anti-aging, hydrating, and anti-inflammatory properties [15].

Also, in terms of cosmetic ingredients, emphasis was placed on using natural, environmentally friendly products. Thus, research has also focused on the extensive use of vegetable oils. These are extremely valuable ingredients in the cosmetic industry. Specialized literature has highlighted that the introduction of vegetable oils into products intended for topical application has beneficial effects, driven by their antioxidant effect, cellular protection, restoration of the skin barrier, as well as the enhancement of the healing process [16]. The present study incorporated vegetable oils, including hemp seed, aloe, centella, pomegranate seed, and calendula, into the newly formulated topical systems.

Hemp seed oil is a powerful anti-inflammatory, helps in wound healing, and moisturizes the skin without clogging pores [17], while pomegranate seed oil is recognized for its skin-regenerating effects [18]. Additionally, oils such as calendula, centella, and aloe, through their compounds, provide benefits to the skin barrier [19,20,21].

In this context, the aim of the present study, through the formulation of honey- and essential oil-based structured systems enriched with other bioactive compounds, was to develop new multicomponent systems with diverse skin benefits.

## 2. Results and Discussion

### 2.1. Organoleptic Properties

Organoleptic assessment of the three emulsions (F1, F2, and F3) was carried out under controlled laboratory conditions, with the results summarized in Table 1 and Figure S1.

### 2.2. Density, pH and Humidity Determination

The density of the three formulations ranged between 0.92 and 0.96 g/cm^3^, which is characteristic of oil-in-water emulsions. The pH values varied between 5.3 and 5.6, indicating good compatibility for topical application, as they fall within the physiological pH range of the skin (Table 2).

The moisture content ranged between 66.22% and 69.05%, with the lowest value observed for F2 and the highest for F3. The obtained data indicated a slight inverse relationship between density and moisture content. Specifically, lower moisture levels corresponded to higher density values, suggesting a more compact internal structure.

### 2.3. Spreadability Assessment

**Figure 1:**
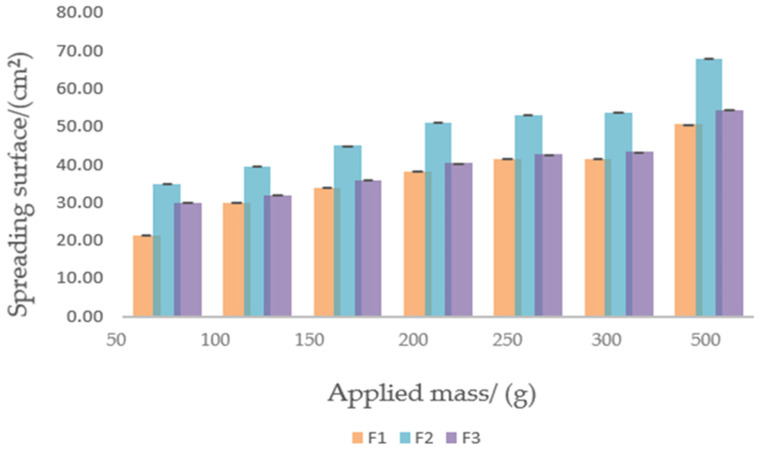
The spreading properties of the emulsion type formulations. Spreadability was expressed as the calculated spreading area (cm^2^) of the circular sample formed between two glass plates under increasing weights.

For the three formulations, the spreadability capacity was analyzed using the extensiometric method. Seven weights were used: 50 g, 100 g, 150 g, 200 g, 250 g, 300 g, and 500 g. The results obtained are shown in Figure 1.

The emulsions tested showed relatively similar behavior throughout the analyses. Of the three formulations, F2 consistently showed the highest spreadability values as the applied weight increased, indicating superior spreadability capacity. This behavior may be advantageous, as it allows for the coverage of a larger surface area using a smaller amount of product.

In contrast, F1 showed the lowest spreadability values, suggesting a more compact structure. This may be beneficial for maintaining prolonged contact with the skin surface after application. F3 showed intermediate values, reflecting a balanced behavior between spreadability and structural consistency.

Overall, all three formulations demonstrated comparable performance, indicating their suitability for topical application, while also exhibiting favorable absorption characteristics.

### 2.4. Rheology Analysis

Rheology analyzes the response of materials to deformation, specifically to applied stresses. Thus, the rheological fingerprints of the formulations reflect both their internal architecture and key properties such as ease of spreading [22,23].

The recorded flow profiles show that the viscosity of the tested formulations decreases with increasing shear rate, indicating a pseudoplastic, shear-thinning, non-Newtonian character.

**Figure 2:**
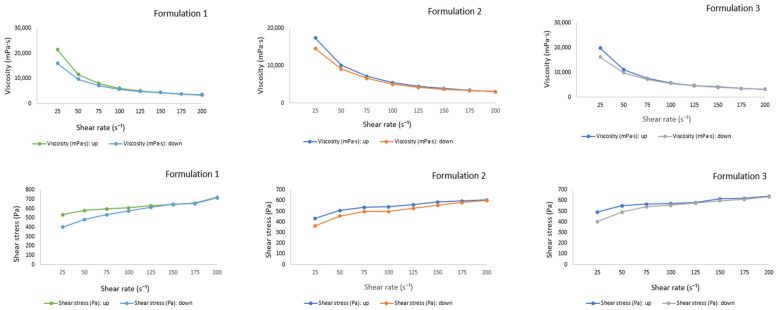
Rheological behavior of formulations F1–F3: viscosity (mPa·s) and shear stress (Pa) as a function of shear rate (s^−1^).

The test results (Figure 2) showed that F1 exhibits pseudoplastic behavior with pronounced thixotropy. The hysteresis is significant, demonstrating slow structural rearrangements that do not recover instantly after the removal of the applied stress. Upon topical application, the product provides an emollient, creamy, “rich” sensation but spreads easily with rubbing.

In contrast, F2 was the most fluid at rest compared to formulations F1 and F3. The system showed pseudoplastic behavior with more moderate hysteresis. Upon topical application, this type of preparation spreads more easily, being light on the skin.

F3 displayed an initial consistency comparable to that of formulation F1. Hysteresis is present but smaller than in F1, suggesting a dense structure, yet with relatively quick rearrangement. Upon application, it spreads easily. Thus, the rheological profiles indicate that the tested formulations exhibit pseudoplastic non-Newtonian flow and shear-thinning behavior, as well as thixotropic characteristics.

### 2.5. GC–MS Analysis

The main volatile components identified by headspace GC-MS in the formulations (F1, F2, F3) and their probable origin are listed in Table 3, Table 4 and Table 5. The complete volatile profiles of the formulations (F1, F2, F3) and raw materials are provided in Supplementary Material, Tables S1–S3. No volatile compounds were detected by headspace GC-MS in the chestnut honey sample (H4) under the experimental conditions employed. Therefore, no chromatographic profile is presented for this sample in the Supplementary Material. The assignment of the major volatile compounds presented in Table 3, Table 4 and Table 5 was based on comparisons with the corresponding essential oil profiles and the analyzed honey samples. To avoid overinterpretation, the term “probable origin” was retained, as the volatile composition of the final formulations may also be influenced by interactions among ingredients and processing conditions.

The headspace GC-MS analysis demonstrated that the characteristic volatile compounds of the incorporated essential oils remained detectable after the emulsification step, indicating their retention within the structured semisolid matrices. The volatile profiles differed considerably among the formulations, reflecting the composition of the incorporated essential oils and the influence of the topical systems on the distribution of volatile compounds.

The results also suggest matrix-related effects on volatile retention and release. Although several volatile compounds were identified in the honey samples, only a limited number remained detectable in the final formulations, possibly due to interactions within the semisolid matrix, reduced volatility after emulsification, or masking by dominant terpene compounds and benzyl alcohol originating from the preservative system.

In addition, the terpene-rich volatile profiles may contribute to the observed skin benefits, including improved hydration, reduced erythema, and enhanced skin surface properties. Oxygenated monoterpenes such as linalool, geraniol, and terpinen-4-ol have been previously associated with antioxidant, soothing, and skin-conditioning properties, which may support the multifunctional behavior of the developed topical systems [24,25].

The identified volatile compounds included bioactive constituents previously associated with antioxidant, anti-inflammatory, antimicrobial, and anti-aging effects in skin.

Geraniol and geranyl acetate are the main volatile compounds in the F1, characteristic components of Palmarosa essential oil, and are associated with anti-inflammatory [26] and antimicrobial properties [27].

A 2022 study highlighted that limonene protects keratinocytes from UVB-induced photoaging by blocking intracellular ROS generation [28].

The second formula, F2 showed a terpene-rich profile dominated by camphene and α-pinene, compounds frequently associated with antioxidant and skin barrier-supportive properties. α-Pinene can protect the skin by reducing stress caused by UVA exposure. It can reduce inflammatory markers such as NF-κB, IL-6, and TNF-α in HaCaT skin cells [29].

F3 was dominated by the lavender-associated volatile profile, especially rich in linalool acetate and linalool, compounds known for their soothing and anti-inflammatory actions [30,31].

### 2.6. In Vivo Assessment of Topical Formulations

#### 2.6.1. Evaluation of Skin pH

A particularly important role in maintaining healthy skin is played by pH. Under non-pathological conditions, the skin’s pH ranges between 4.1 and 5.8. This slightly acidic environment is known as the “acid mantle” and ensures the homeostasis of the stratum corneum and can contribute to antimicrobial defense [32,33,34].

**Figure 3:**
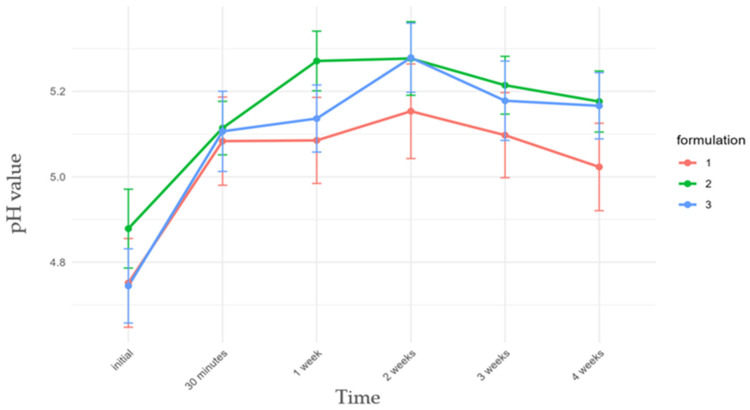
Evolution of skin pH over time for formulations F1–F3. Values are presented as mean ± SE.

The skin’s pH can be influenced by a multitude of factors such as age, pathological condition, and even certain cosmetic products. For this reason, evaluating how topical formulations influence skin pH is an essential step. In this way, it can be assessed whether the products are safe for topical use and whether they have an appropriate safety and tolerability profile for cutaneous application (Figure 3) [32].

For all three formulations tested at the topical level, small-magnitude changes (0.02–0.71) were observed, which were nevertheless statistically significant. Over time, from the baseline, pH values showed a significant effect (*p* < 0.001, η^2^p = 0.677), indicating that pH varied throughout the monitoring period. Most volunteers had a slightly acidic initial skin pH, with values showing a slight increase that did not, however, disrupt the integrity of the skin barrier. In contrast, no significant differences were observed between formulations (*p* = 0.062, η^2^p = 0.189) or in the evolution of pH over time between formulations (*p* = 0.140, η^2^p = 0.104), suggesting a similar behavior of all analyzed systems. Minor variations were observed among volunteers that fall within the normal biological variability. For example, volunteer 11 (31 years old) showed one of the largest increases in pH values for F2, of 0.59 units, from 4.66 to 5.25. These variations did not influence the overall trend.

Thus, the results obtained indicate that the three formulations only cause slight increases in skin pH, without disrupting the physiological balance of the skin, which ensures tolerance during use.

#### 2.6.2. Evaluation of Skin Hydration (Corneometer)

When formulating products for topical application, their moisturizing capacity is an essential characteristic because healthy skin is associated with well-hydrated skin. An indispensable component for the proper functioning of the skin is water. Water plays an important role in maintaining the normal function of the skin. At the level of the stratum corneum, there are corneocytes interconnected by a lipid matrix mainly composed of fatty acids, ceramides, and cholesterol. This structure forms the skin barrier, which serves to prevent excessive transepidermal water loss [35,36].

Also, at the level of the stratum corneum are osmolytes, which are chemical compounds such as lactic acid, sugars, and free amino acids. By binding to water molecules, osmolytes contribute to preventing excessive water loss, ensuring that the skin maintains optimal moisture [35].

The formulations in this study, F1, F2, and F3, contain honey in their composition. The specialized literature reports that honey is used at concentrations ranging from 0.5% to 5% in creams and emulsions [37]. Products containing honey contribute to increased skin hydration and improved skin barrier properties due to the presence of sugars. These sugars have the ability to bind water molecules, thereby maintaining an optimal level of hydration in the stratum corneum. For the present research, each formulation contains 5% honey. Additionally, formulations that contain both honey and glycerin have demonstrated enhanced moisturizing effects due to the humectant properties of glycerin [37,38]. Thus, all three formulations in this study include both honey and glycerin in their composition. Furthermore, recent research has highlighted that Aquaporin-3 (AQP3), a transmembrane protein involved in water transport at the cellular level, is also involved in the transport of glycerin to the stratum corneum. At the cutaneous level, AQP3 is predominantly expressed in keratinocytes. Studies have shown that a reduction in glycerin concentration in the epidermis is associated with decreased water content and skin elasticity [39].

**Figure 4:**
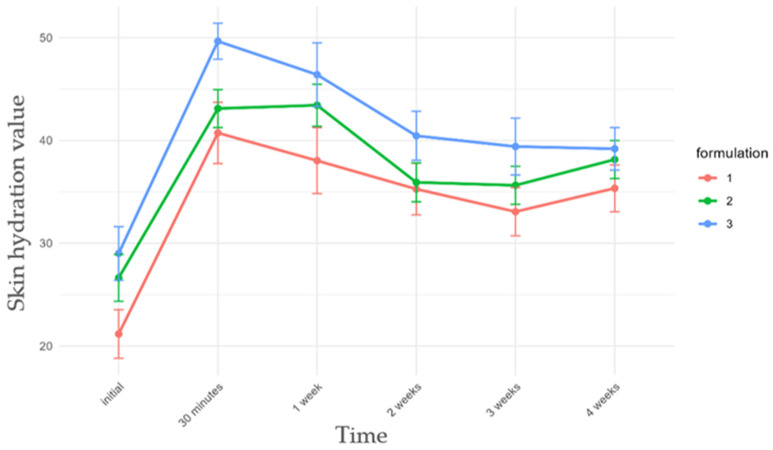
Evolution of skin hydration over time for formulations F1–F3. Values are presented as mean ± SE.

Moreover, F3 contained 0.4% ceramides in contrast to the other two. In Figure 4, it can be observed that F3 exhibited higher skin hydration values compared to formulations F1 and F2. This trend can be associated with the presence of ceramides that help maintain the integrity of the skin barrier and reduce transepidermal water loss [40]. Corneometry is a method used in dermatological studies to evaluate skin hydration levels [35].

Through corneometer testing, it was observed that for the three formulations analyzed, there was an increase in the recorded values over time; this increase was statistically significant (*p* < 0.001, η^2^p = 0.673). The most pronounced increase in values was observed between the initial time point and 30 min after application; thereafter, a slight decrease occurred, but it still exceeded the initial measurement. Thus, skin hydration remained constant throughout the application period.

Comparing the formulations, there are significant differences between them (*p* = 0.007, η^2^p = 0.318), with values generally being higher for F3, followed by F2 and F1. However, the changes over time followed a similar trend for all formulations (*p* = 0.580).

According to the post hoc analysis, the significant differences were between the initial time point and subsequent measurements (initial—30 min, initial—1 week, initial—2 weeks, initial—3 weeks, initial—4 weeks). There were no significant differences between subsequent time intervals (e.g., between 1 week and 2 weeks, between 2 weeks and 3 weeks).

The trend was an increase in skin hydration, with more pronounced effects for some subjects. For example, for F1, subject no. 15 (age 47, dry skin, phototype II) showed significant increases compared to baseline, which were maintained throughout the study (baseline: 14.2, 30 min: 49.16, 1 week: 50.70, 2 weeks: 50.34, 3 weeks: 45.10, 4 weeks: 43.92). For F2, in the case of subject no. 13 (age 47, normal skin, phototype II), a marked increase was recorded between baseline and 30 min after application, with values rising from 5.78 to 42.82, representing a strong response also explained by the high level of dehydration of the skin. For F3, in the case of subject no. 8 (age 52, combination skin phototype II), it was observed that the hydration level, compared to baseline, doubled from 16.64 to 32.46, a trend that persisted for all test time points.

After this period, the values stabilize, indicating both a rapid effect of the three formulations and their maintenance over time.

#### 2.6.3. Evaluation of Skin Melanin Content

The skin color is mainly attributed to melanin, a component synthesized by melanocytes. Melanin plays a particularly protective role against oxidative stress and ultraviolet radiation. Melanogenesis, the process of melanin synthesis, can be influenced both by endogenous factors, such as hormonal status or genetic factors, and by exogenous factors, such as oxidative stress, exposure to UV radiation, or the use of topical substances, such as hydroquinone, kojic acid, and azelaic acid [41,42].

**Figure 5:**
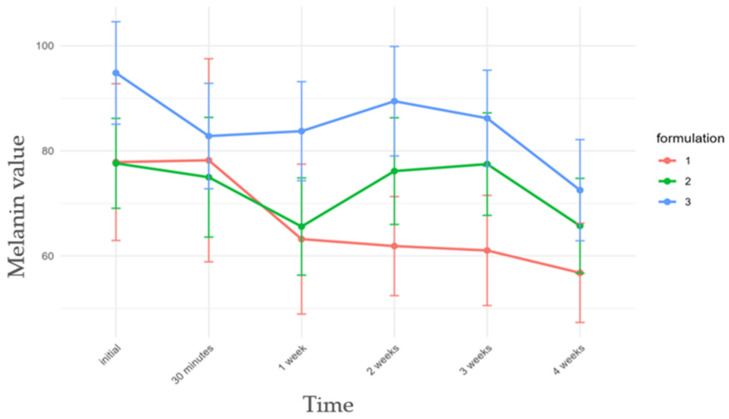
Evolution of melanin values over time for formulations F1–F3. Values are presented as mean ± SE.

In the analysis of the melanin parameter for all three formulations applied during the testing period, no significant changes over time were observed (*p* = 0.127, η^2^p = 0.115). Figure 5 shows that the three formulations caused slight variations in skin pigmentation, which are not statistically relevant. This may suggest that the observed changes fall within normal physiological variability, without indicating a depigmenting effect.

Post hoc analysis further confirmed the absence of significant differences between the initial time point and the other tested intervals.

Thus, the evaluation of this parameter suggests that the three formulations do not significantly influence skin melanin levels. It can be inferred that the tested products are well tolerated by the skin, without inducing depigmenting or hyperpigmenting effects.

#### 2.6.4. Evaluation of Skin Erythema

**Figure 6:**
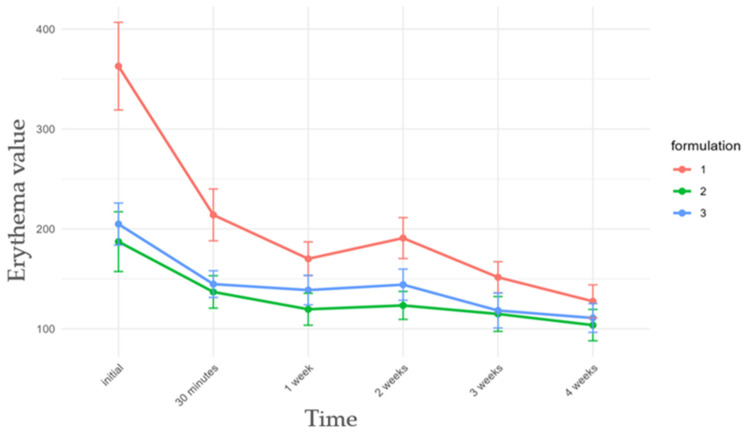
Evolution of erythema values over time for formulations F1–F3. Values are presented as mean ± SE.

Erythema is a simple increase in blood flow at the skin level, causing dilation of the dermal vessels, which gives the skin a red appearance. A multitude of factors can lead to the appearance of erythema, such as fever, warm environments, infections, or acne. In studies, the reduction in erythema is considered an indicator of improvement in skin condition [43,44]. For the volunteers enrolled in the present research, a decrease in erythema was recorded (Figure 6).

Erythema decreased over the testing period for all the products tested. The effect is significant (*p* < 0.001, η^2^p = 0.665). The most pronounced decrease was observed between the initial time and 30 min. The reduction was maintained throughout the study period. For example, in the case of subject no. 9 (age 67, dry skin, phototype II), 30 min after the application of F1, the item value dropped almost by half, from 232.6 to 151.8; the decreasing trend was maintained throughout the analysis period, reaching a value of 62.4 (at 4 weeks). Also, for product 3, the results were similar; for example, subject no. 10 (age 51, combination skin, phototype II) had a significant reduction in erythema 30 min after application (from 300.4 to 118.8). After 4 weeks, the value was 87.8.

There were differences between the formulations (*p* < 0.001, η^2^p = 0.484), as well as in the temporal evolution of erythema between them (*p* = 0.012, η^2^p = 0.256). Formulations 2 and 3 had lower and more stable values compared to Formulation 1.

In the post hoc analysis, significant differences were observed between baseline and subsequent determinations. The results indicate a decrease in erythema following the application of the formulations, suggesting good tolerability over time, as well as a soothing effect.

A key ingredient responsible for this effect may be honey. It is a compound for which efficacy has been demonstrated in treating eczema, cuts, burns, wounds, and inflammatory skin conditions. Studies have shown that honey can modulate the immune response at the skin level and can reduce excessive inflammation by decreasing the production of certain molecules involved in oxidative stress and inflammatory processes, contributing to the amelioration of local inflammation [45,46].

In addition to the other components of the formulation F1, hemp seed oil and aloe are also included. Bioactive compounds derived from hemp are associated in the literature with calming, anti-inflammatory, and moisturizing effects [47], while aloe vera oil is characterized by the presence of bioactive compounds, such as polysaccharides, which have anti-inflammatory properties and support skin repair [48].

In F2, the combination of aloe oil with pomegranate seed oil may suggest a stable erythema-reducing effect during testing. Pomegranate seed oil is rich in punicic acid and lipid compounds that play a protective role on the skin barrier [49].

In F3, the effect of reducing erythema may be associated with calendula oil and *Centella asiatica* oil. Studies have shown that *Calendula officinalis*, through its active compounds, has healing and anti-inflammatory effects [50], while *Centella asiatica* is associated with supporting skin repair processes [51].

Thus, the combination of honey, vegetable oils, and the other components in the formulation may contribute to supporting the skin barrier and alleviating erythema.

#### 2.6.5. Evaluation of Transepidermal Water Loss (TEWL)

Transepidermal water loss (TEWL) refers to the water lost through the stratum corneum. The more impaired the skin barrier, the higher the TEWL values. TEWL values can vary depending on numerous factors, such as age, pathological condition of the skin, or the anatomical area analyzed [52,53,54].

**Figure 7:**
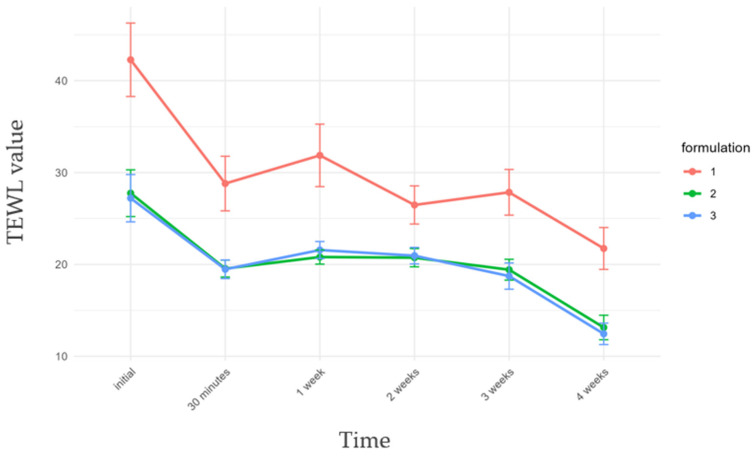
Evolution of transepidermal water loss (TEWL) values over time for formulations F1–F3. Values are presented as mean ± SE.

In the testing of the TEWL parameter, a progressive decrease was recorded over the course of four weeks, with a significant effect over time (*p* < 0.001, η^2^p = 0.601). Although there is a significant difference between the formulations (*p* < 0.001, η^2^p = 0.642), as well as in the evolution of TEWL over time between them (*p* = 0.029, η^2^p = 0.176), the trend is the same, namely a decrease in the values recorded during the analysis. For example, for F2, subject no. 4 (age 24, dry skin, phototype II) experienced a decrease of more than half compared to the baseline, from 26.27 to 11.13. The highest values were observed with F1, but the initial values were also higher, indicating that at the start of the testing, the subjects had a more impaired skin barrier (Figure 7).

Therefore, for all three obtained formulations, a reduction in cutaneous water loss is indicated, which reflects an improvement in the skin barrier.

#### 2.6.6. Evaluation of Skin Elasticity (Cutometer)

The Cutometer is a standard non-invasive instrument used to measure skin elasticity. This study focused on the analysis of the R5 parameter. The R5 parameter represents the net elasticity of the skin, with higher values indicating greater cutaneous elasticity. As a dimensionless parameter independent of skin thickness, R5 shows better reproducibility compared to dimensional parameters such as R1, R3, and R4. Furthermore, R5 may contribute to a better understanding of in vivo skin elasticity [55,56,57].

**Figure 8:**
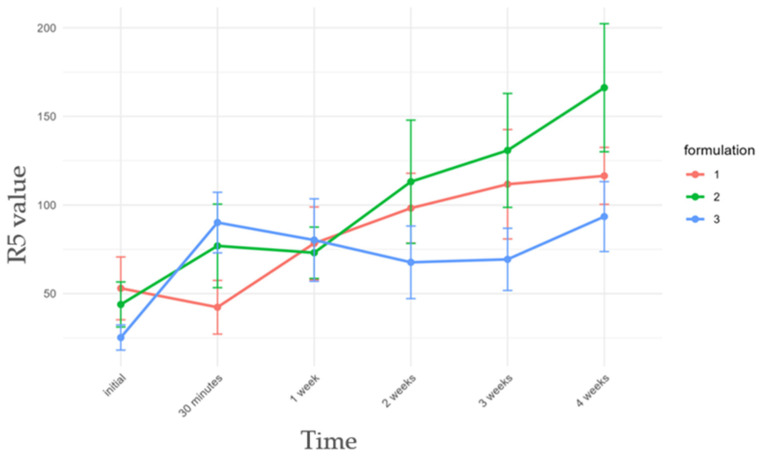
Evolution of cutometer parameter R5 values over time for formulations F1–F3. Values are presented as mean ± SE.

In Figure 8, it is observed that over time the values show an increasing trend, a trend that, from a statistical point of view, has a significant effect (*p* = 0.004, η^2^p = 0.235). There are no significantly different values between formulations (*p* = 0.189, η^2^p = 0.107). In the post hoc analysis, the differences are limited for F1 and F3; in contrast, a significant difference was observed between the initial time point and 4 weeks for F2 (*p* = 0.0015). The study showed that the enrolled subjects experienced a moderate improvement in skin elasticity, statistically evident for F2.

#### 2.6.7. Evaluation of Skin Firmness (Indentometer)

The indentometer is a device whose operating principle consists of using a force to deform the skin, measuring the depth of penetration at the cutaneous level. A decrease in the values may indicate that the skin has become more resistant to deformation, which translates into an improvement in firmness [58].

**Figure 9:**
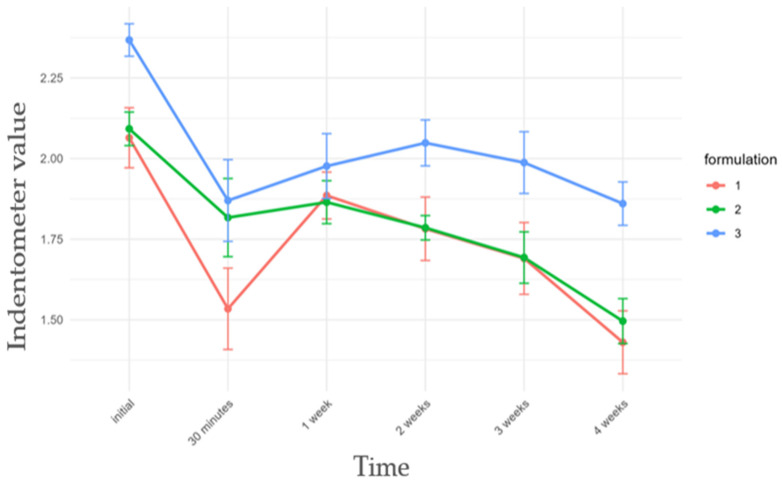
Evolution of indentometer values over time for formulations F1–F3. Values are presented as mean ± SE.

Indentometer testing showed significant changes over time (*p* < 0.001, η^2^p = 0.425). Significant differences were observed between formulations (*p* = 0.002, η^2^p = 0.414). The trend for all formulas was the same, namely a gradual decrease, more noticeable towards the end of the testing period. The device recorded a decrease in skin deformability, which indicates an increase in its firmness. Post hoc analysis highlights significant differences, particularly between the initial time point and the evaluation at the end of the study (4 weeks). Thus, the results indicate an improvement in skin firmness (Figure 9).

#### 2.6.8. Evaluation of Skin Topography

For the topographical analysis of the skin, the Visioscan^®^ VC 20plus system (Courage + Khazaka Electronic GmbH) was used. The device operates on an optical principle with UV-A light, with a peak emission at 390 nm. When the skin is dry, the corneocytes emit specific fluorescence, resulting in images with brighter areas (white pixels). Hydrated skin will have reduced light reflection, and the generated images will be more uniform and darker. An important element of the analysis is the 3D images. These three-dimensional representations facilitate the visualization of differences in ridge elevations and topographical furrow depths [59].

The SELS parameters (Surface Evaluation of the Living Skin) are represented by SEr (roughness), SEsc (scaliness), SEsm (smoothness), and SEw (wrinkles). SEr evaluates the overall roughness of the image. SEsc reflects the degree of desquamation of the stratum corneum, so lower values of this parameter indicate less desquamated skin. SEsm indicates the degree of skin smoothness. Smooth and uniform skin presents a smaller variety of gray tones, so lower SEsm values are associated with smoother skin. Regarding the SEw parameter, the higher its value, the more numerous, wider, and deeper the wrinkles are [54,59].

In the statistical analysis of the SELS parameters, it was observed that for all of them, significant changes over time were recorded (*p* < 0.001). In the case of SEr (roughness), although the ANOVA analysis highlighted a significant effect of time, post hoc analyses identified significant differences for F2 and F3. For F1, no significant changes were observed between the initial measurement and 4 weeks (*p* = 0.22). Post hoc analysis for SEsc showed statistically significant changes, especially between the initial measurement and the end of the testing period. Thus, the tested emulsions led to a progressive reduction in this parameter. Lower SEsc results suggest reduced scaling.

**Figure 10:**
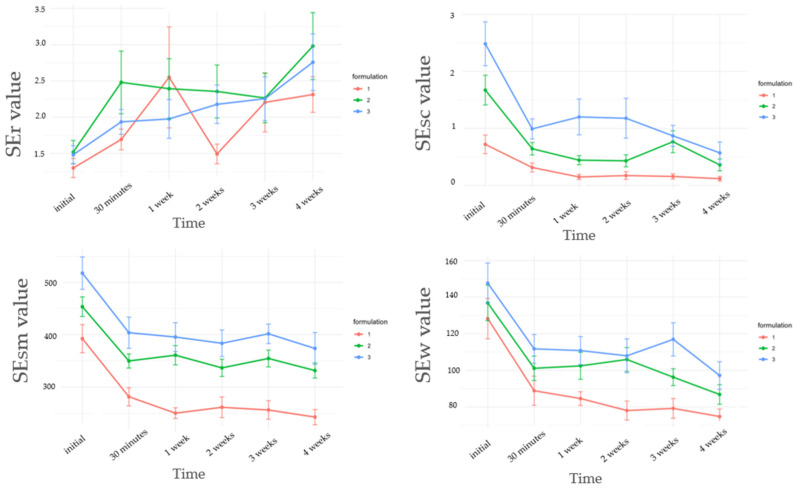
Evolution of SELS parameters over time for formulations F1–F3: SEr (skin roughness), SEsc (skin scaliness), SEsm (skin smoothness), and SEw (skin wrinkles). Values are presented as mean ± SE.

For the parameters SEsm and SEw, significant changes were observed from the start of testing to the end of the study (4 weeks). The trend of these parameters was downward throughout the testing period (Figure 10). Thus, the results indicate that all formulations improved skin smoothness and reduced wrinkles.

The analyzed surface parameters are the area and the volume of the skin surface. Over time, after topical treatment, the surface value should show a decreasing trend [59]. The values of the surface index are significantly affected by time (*p* < 0.001, η^2^p = 0.584); the trend is decreasing. Lower observed values of the skin surface are associated with better skin quality. Post hoc analysis revealed significant differences between the start of testing and the evaluations carried out at 2, 3, and 4 weeks for all formulations (*p* < 0.05).

**Figure 11:**
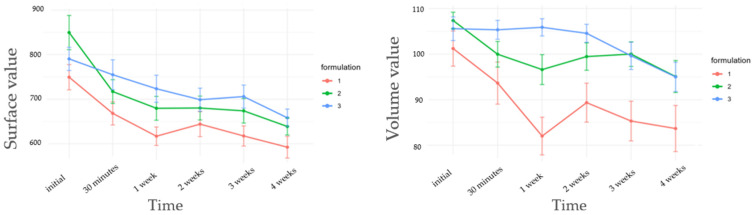
Evolution of surface parameters over time for formulations F1–F3: surface and volume. Values are presented as mean ± SE.

For the volume parameter, the effect of time was also statistically significant (*p* = 0.002, η^2^p = 0.261), with the values showing a decreasing trend over time (Figure 11). Post hoc analysis showed significant differences between the initial measurement and subsequent evaluations for F1 (*p* < 0.05). For formulations F2 and F3, although a decreasing trend over time was observed, the differences were not statistically significant (*p* > 0.05).

The results obtained from the analysis of textural parameters (contrast, entropy, variance, energy, and homogeneity) showed a significant influence in relation to time on the variables with *p* < 0.001. The contrast parameter showed a decreasing trend for all three formulations. The reduction in contrast indicates a good condition of the skin. Similarly, variance exhibited a decreasing trend over the course of the study period. The decrease in this parameter reflects a less rough skin texture [59]. The entropy parameter gradually increased over time for all three formulations. The increase in entropy may suggest more hydrated skin [54]. Additionally, the energy and homogeneity parameters progressively increased throughout the study, which may be associated with younger skin and a uniform appearance [59].

**Figure 12:**
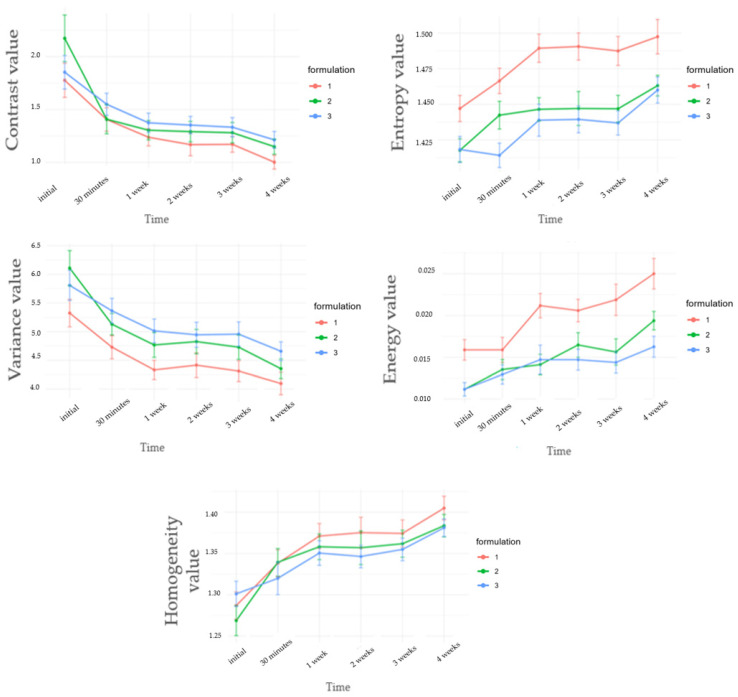
Evolution of textural parameters over time for formulations F1–F3: contrast, entropy, variance, energy, and homogeneity. Values are presented as mean ± SE.

Post hoc analyses showed that the majority of statistically significant differences were observed between baseline and the 3–4-week testing for all three formulations. Additionally, the observed trends of decreasing contrast and variance, concomitant with increasing entropy, energy, and homogeneity, may suggest wrinkle improvement along with the uniformization of its surface (Figure 12).

**Figure 13:**
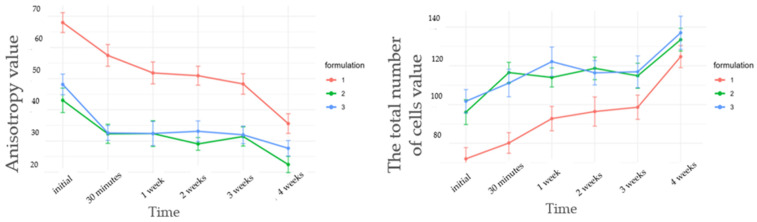
Evolution of skin microrelief parameters over time for formulations F1–F3: anisotropy index and total cells. Values are presented as mean ± SE.

Aging parameters include the anisotropy index (directionality of the lines) and the total number of cells. A low anisotropy index is associated with younger skin, as well as with uniformity of skin lines. Thus, an increased index suggests the opposite, often being associated with older-appearing skin. Regarding the other parameter, an increase in the number of cells indicates skin with higher cell density and a younger appearance [54]. The present study recorded a decrease in the anisotropy index and an increase in the total number of cells. From a statistical standpoint, the analysis of both the anisotropy index and the total number of cells recorded a significant effect of time for both (*p* < 0.001). The most pronounced decreases in the anisotropy index were observed for F2 and F3 at the end of the experimental period. At the same time, the highest values at 4 weeks were also detected for F2 and F3 for the total number of cells (Figure 13).

Post hoc analyses for the two parameters highlighted significant differences, particularly between the baseline and the assessments conducted after 3 and 4 weeks for all three formulations.

**Figure 14:**
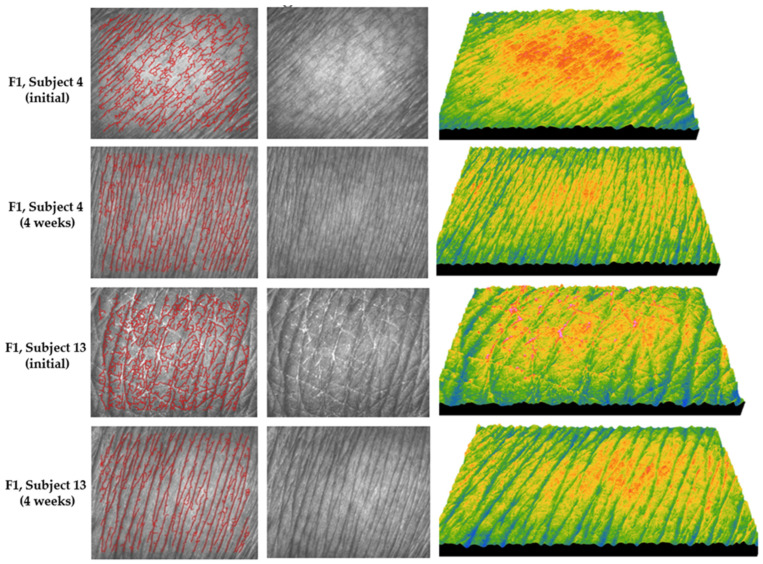
Representative images of skin topography recorded for formula F1 in subject no. 4 (age 24) and subject no. 13 (age 47) at baseline and after 4 weeks of treatment; the images include the wrinkle line map (**left**), grayscale images of the skin surface (**middle**), and 3D images of the skin microrelief (**right**).

In Figure 14, the topographical changes for two subjects at two important testing moments can be observed, initially and 4 weeks after the application of formula 1. The black-and-white images capture the microrelief of the skin through the lines. At the initial testing moment, both subject no. 4 (age 24) and subject no. 13 (age 47) present numerous bright areas on the skin, which may suggest lower hydration of the skin layer. Additionally, the lines are unevenly distributed and interrupted. At the end of the 4 weeks, the disappearance of these highly bright areas is observed for both subjects, which may suggest that the formulation improved and homogenized the skin microrelief. The lines are more uniform and better organized.

The 3D color images show the differences in height and roughness of the skin surface. For subject no. 4, at the time of initial testing, in the 3D color image, yellow-orange areas predominate, alongside red-associated areas. Those central red-brown areas are the peaks of the skin that may suggest the presence of bumps or unevenness. The yellow areas surrounding the orange center have lower heights, with medium roughness. The green zones represent smooth portions without pronounced bumps or wrinkles. The image at the end of the study (4 weeks) shows a reduction in the yellow-orange zones with an increase in the green ones. This aspect suggests a smoother skin appearance, free of pronounced unevenness. For subject no. 13 as well, the initial 3D image shows reddish prominences and some bluish coloration. The blue areas may indicate the presence of more pronounced folds or areas with reduced hydration. In the four-week testing, the presence of reddish and white areas is reduced, indicating more uniform and hydrated skin.

**Figure 15:**
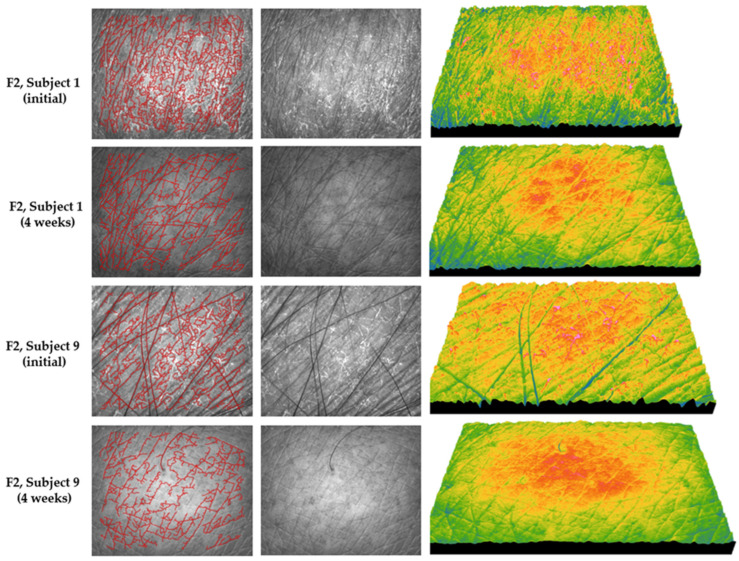
Representative images of skin topography recorded for formula F2 in subject no. 1 (age 87) and subject no. 9 (age 67) at baseline and after 4 weeks of treatment; the images include the wrinkle line map (**left**), grayscale images of the skin surface (**middle**), and 3D images of the skin microrelief (**right**).

The evolution of the application of F2 for subjects no. 1 (age 87) and no. 9 (age 67) is highlighted in Figure 15. For subject no. 1, in the black-and-white images, the presence of numerous very bright, highly reflective areas with an interrupted, uneven distribution of lines can be observed. These indicate non-homogeneous areas with possible insufficient hydration. Testing at the end of the study, after the application of F2, in the black-and-white images, shows that the lines become more uniform, with better organization. The bright areas are also reduced, which indicates an improvement in the skin’s appearance. In the 3D images, the initial red-orange areas are reduced, with a decrease in contrast between elevated and recessed areas, suggesting an improved skin texture. In the case of subject no. 9, the initial black-and-white images highlight numerous irregular lines and highly bright areas. Hair strands accentuate the heterogeneous appearance.

In 3D, the images are dominated by yellow-orange areas with red-brown regions. At the end of the study, the black-and-white images show a reduction in bright areas, while the 3D reconstruction records an increase in green and yellow areas, indicating a more uniform surface. Thus, the use of F2 in the case of the examples presented over the course of the 4 weeks led to a progressive improvement of the skin microrelief, with a more uniform distribution and organization of the skin lines (Figure 15).

**Figure 16:**
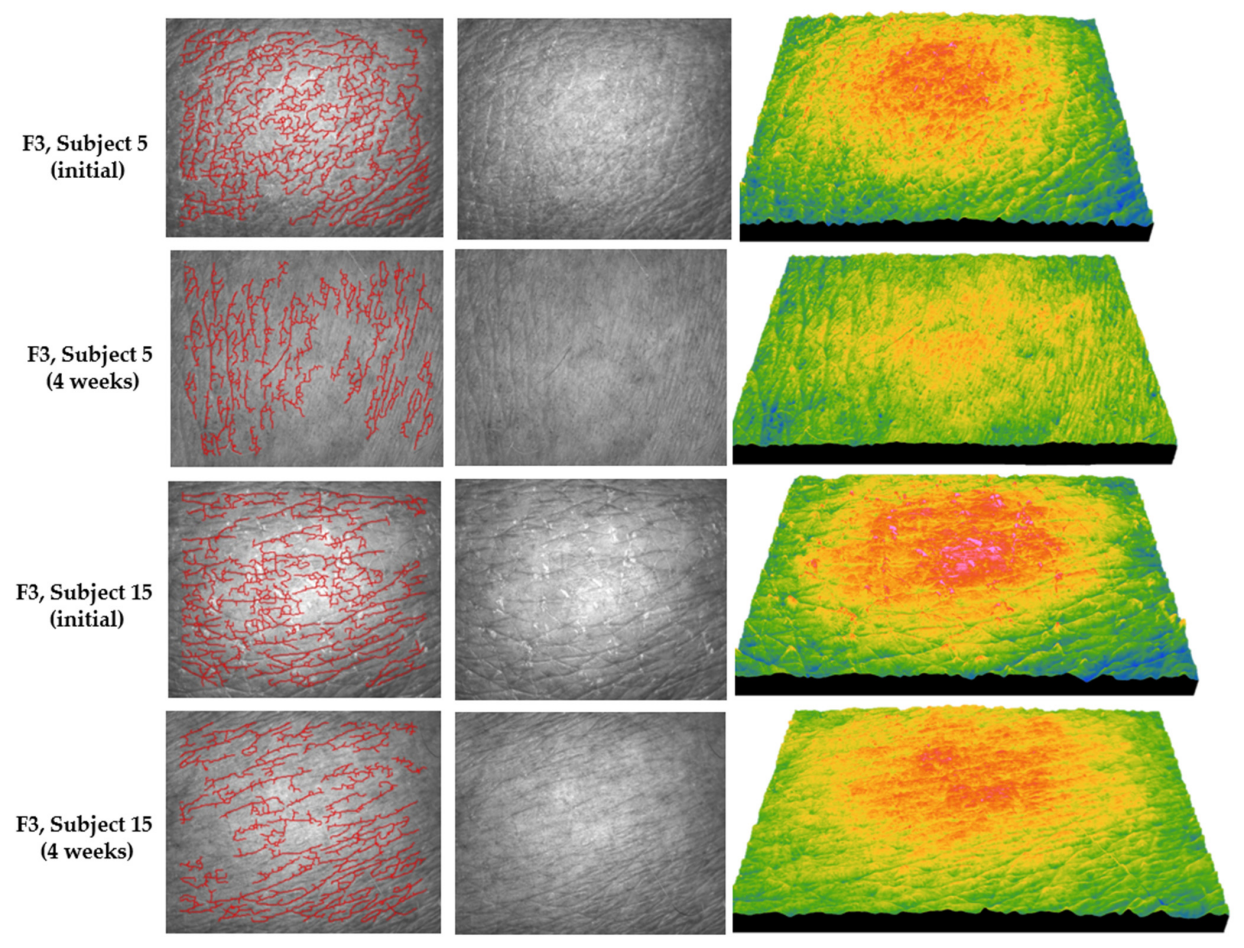
Representative images of skin topography recorded for formula F3 in subject no. 5 (age 26) and subject no. 15 (age 47) at baseline and after 4 weeks of treatment; the images include the wrinkle line map (**left**), grayscale images of the skin surface (**middle**), and 3D images of the skin microrelief (**right**).

For F3, the black-and-white and 3D images are presented in Figure 16. For both subject no. 5 (age 26) and subject no. 15 (age 47), in the images taken at the beginning of the testing, highly luminous areas can be observed, which may be associated with dehydrated skin. After 4 weeks, these areas appear considerably reduced and improved. For subject no. 5, the initial 3D image highlights skin relief with highly red-orange areas, corresponding to pronounced irregularities. At the end of the study, following proper application of F3, the intensely colored areas are reduced, with the red areas almost completely eliminated, suggesting an overall smoothing of the skin surface. A similar result is observed in the case of subject no. 15.

Thus, the black-and-white and 3D images obtained for all three formulations demonstrated an improvement in skin appearance after the 4-week period. The microrelief lines have become more homogeneous, and the orange-red areas have been significantly reduced.

The beneficial effects observed over the 30-day testing period can be associated with the presence of the components selected for the formulations.

The increase in values measured with the corneometer suggests an improvement in skin hydration, an effect that can be associated with components such as honey, glycerol [37,39], and hyaluronic acid [15].

Additionally, during testing, a reduction in TEWL values was observed for all three formulations, demonstrating an improvement in the skin barrier and a decrease in transepidermal water loss. Ceramides play a role in maintaining skin barrier integrity [34]. At the same time, the plant oils used, such as hemp seed oil [47], aloe [48], pomegranate seed [49], calendula [50], and *Centella asiatica* [51], are associated with protective effects on the skin.

The decrease in parameters such as SEw wrinkles, variance, anisotropy index suggests a rejuvenating effect at the skin level. These effects may be due to the presence of coenzyme Q10, vitamin E, and niacinamide. Vitamin E is known for its antioxidant action, protection against oxidative stress, and improvement of elasticity [60,61]. Meanwhile, coenzyme Q10 reduces signs associated with skin aging [62], and the reduction in erythema values may be associated with the anti-inflammatory properties of honey, bisabolol [14], niacinamide [61], as well as vegetable oils such as calendula [50], aloe [48], and *Centella asiatica* [51]. Additionally, the increase in the evaluated R5 elasticity parameter may indicate an improvement in elasticity, and allantoin can stimulate collagen synthesis [13].

The development of multi-component structured emulsions containing multiple types of honey, essential oils, and vegetable oils represents a shift from traditional reductionist formulation screening toward holistic, systems-oriented dermatocosmetic design [63]. In phytotherapy and advanced cosmetics, natural raw materials are intrinsically complex chemical products; for example, honeys provide a rich matrix of organic acids, enzymes, and hygroscopic oligosaccharides, while essential oils contribute localized volatile monoterpenols [64]. Rather than functioning as isolated additives, these components may act through a multi-target mechanism, simultaneously addressing different physiological layers of the skin barrier and dermal matrix.

Evaluating these systems by adding single ingredients step by step may not fully capture the interfacial competitive behavior and microrheological integration that occur within the finished structured matrix. Empirical evidence supporting distinct, composition-specific synergies is directly reflected in the divergent in vivo clinical data of this study. Despite sharing an identical O/W base architecture, Formulation F3 exhibited superior performance as an advanced hydro-retentive, barrier-supportive platform, whereas Formulation F1 showed greater improvements as an anti-aging, texturizing intervention [65,66].

These findings support the concept of systems-level evidence of synergy. It demonstrates that the specific combination of bioactives in each formulation cross-modulates their individual behaviors, yielding a unified final product with targeted clinical specificity that could not be achieved with a simplified, single-active vehicle [63].

A critical evaluation of the experimental data reveals a pronounced composition-effect divergence between the two most functionally distinct structured systems, F1 and F3. Although both formulations share the same structural O/W emulsion framework, their specific bioactive configurations (comprising distinct honey duos, vegetable oils, and essential oils) led them to follow completely different clinical micropathways during the 4-week in vivo topical study.

F3 emerged as the premium moisturizing and barrier-support system, showing the most pronounced increase in skin hydration and the steepest decline in transepidermal water loss (TEWL). From a compositional standpoint, this performance is driven by a highly efficient humectant–occlusive synergy. The honey types selected for F3 (such as manna or Tualang profiles) are rich in hygroscopic sugars, particularly fructose and glucose. When applied topically, these saccharides form a dense network of hydrogen bonds that trap and retain water molecules directly within the stratum corneum [45,67]. This effect, observed in the case of F3, can also be attributed to the synergistic action of its moisturizing components from honey, together with NP ceramide. Ceramides play a key role in maintaining moisture at the stratum corneum [68]. Conversely, Formulation F1 showed a superior capacity to optimize skin surface microtopography, yielding notable improvements in skin textural uniformity, roughness parameters, and wrinkle-related features. This distinct anti-aging and texturizing performance is directly correlated with the advanced antioxidant and regenerative cascade of its specific phytochemical load. One of the essential oils from F1 (such as palmarosa) possesses antioxidant properties [69,70,71,72]. At the same time, F1’s vegetable oil phase, particularly hemp seed oil, rich in essential fatty acids, tocopherols, and phytosterols, may have contributed to the observed improvements in skin texture [47,73]. Combined with the enzymatic, organic acid, and antioxidant profile of its Manuka and Tualang honey pair, F1 may have further contributed to the observed improvements in skin texture [74,75,76].

In summary, this targeted composition-effect analysis shows that the structured systems developed are not generic emollient matrices. Instead, they represent customizable dermo-cosmetic products in which precise botanical architecture dictates clinical specificity: F3 acts as an optimal barrier-repair and hydro-retentive system, while F1 functions as a potent texturizing and anti-aging intervention.

## 3. Materials and Methods

### 3.1. Materials

In the present study, three oil-in-water emulsion formulations were developed, each containing 5% honey. The formulations F1, F2, and F3 were designed with bioactive compounds intended to support hydration and improve the microrelief and appearance of the skin.

The three topical structured systems were prepared using ingredients provided by Agricola Total SRL (Bucharest, Romania) (distilled water), Elemental SRL (Oradea, Romania) (glycerin, propanediol, hyaluronic acid LMW, aloe oil, hemp seed oil, calendula oil, Centella oil, niacinamide, panthenol, allantoin, bisabolol, coenzyme Q10, Ceramide NP, cistus essential oil, palmarosa essential oil, lavender essential oil, and Cosgard), NaturallHome (Salonta, Romania) (Olivem 1000, cetyl alcohol, deodorized cocoa butter, and vitamin E), Aliver (Apex CE Limited, Co. Cork, Ireland) (pomegranate seed oil), Making Cosmetics (Redmond, WA, USA) (cholesterol) as well as Manuka honey (New Zealand), supplied by NutriZing (London, UK), Tualang honey (Malaysia), supplied by Health Harvest Food Limited (Wanchai, Hong Kong), chestnut honey (Spain), supplied by Apicola Pastorul Georgescu S.R.L. (Jilava, Romania), and manna honey (Romania), supplied by a local beekeeper.

### 3.2. Formulation of Topical Structured Systems

The formulations presented in this study represent the final compositions selected after several preliminary formulation trials. The composition was iteratively refined to achieve physically stable semisolid oil-in-water systems with appropriate rheological behavior and suitability for topical application.

The composition of the formulations developed within the study is presented in Table 6. The new topical structured systems are oil-in-water (O/W) emulsions, containing various types of honey, bioactive vegetable oils, essential oils, as well as other compounds. The components of the formulations can be divided into three technological phases: the aqueous phase, the fatty phase, and the cooling phase.

### 3.3. Methods

In the formulation preparation stage, the hydrophilic components of phase A were mixed and heated to 60–70 degrees. Separately, the lipophilic components of phase B were weighed, placed in a capsule, and heated in a water bath at 80 °C until completely melted, yielding a homogeneous mixture. Subsequently, the aqueous phase was added to the oily phase and homogenized using an automatic mixer FagronLab™ PRO (Fagron, Rotterdam, The Netherlands) at 600 rpm for 1 min and 33 s, until a homogeneous emulsion was achieved. After cooling the emulsion to a temperature below 40 °C, the ingredients of phase C (essential oils, vitamin E, bisabolol, the preservative, as well as the other ingredients listed in Table 6) were incorporated, and the formulation was homogenized again at 800 rpm for 1 min and 33 s, until a uniform and stable emulsion was obtained. Each formulation was prepared in batches of approximately 200 g. The final products were packaged in tightly closed containers and labeled accordingly (Figure S2).

#### 3.3.1. Physicochemical Determination

In order to create a profile of the properties of emulsions, such as texture, color, and odor, physicochemical tests were conducted in the laboratory, adhering to standard analysis conditions.

*Density, pH and Humidity Determination*

The density was determined by weighing and measuring the volume for each formulation. The ratio between mass and volume was calculated to determine the density.

For the determination of pH, a calibrated ExStik pH meter (PH110) (Extech, Taiwan) was used.

The moisture content of the samples was assessed by determining the loss on drying, using a thermogravimetric method. Experimental measurements were performed using a Mettler Toledo HR 73 halogen moisture analyzer (Mettler-Toledo GmbH, Greifensee, Switzerland), a device that allows precise and automated monitoring of sample mass variations during the heating process [77].

*Spreadability Study*

The determination of the spreadability of the three formulations was carried out using the Ojeda–Arbussa method, a standardized technique used for evaluating the spreading surface of semisolid systems under controlled weights. For the determination, 1 g of each formulation was taken and placed on a glass plate. Subsequently, a second glass plate weighing 149.3 g was applied on top of the sample. The diameter resulting under this weight was measured. Thereafter, additional weights of 50 g, 100 g, 150 g, 200 g, and 500 g were placed on the upper plate. The weights were applied successively; the diameter was recorded after an individual rest of 60 s for each plate. The spreading surface of each formulation was mathematically calculated based on the measured diameter [78].

The spreading area (*S*) was calculated from the measured diameter (*d*) of the circular sample formed between the two glass plates using the equation:

$$S=\pi {\left(\frac{d}{2}\right)}^{2}$$

where *S* is the spreading area (cm^2^), and *d* is the measured diameter (cm).

*Rheology*

For the rheological determinations, a rotational viscometer (B-One Plus, Lamy Rheology, Champagne-au-Mont-d’Or, France) equipped with the TE-96 spindle (AMETEK Brookfield, Middleboro, MA, USA)was used. For each formulation, a two-step testing protocol was applied, ascending from 25 s^−1^ to 200 s^−1^ and descending from 200 s^−1^ to 25 s^−1^. Eight shear rate levels (25, 50, 75, 100, 125, 150, 175, 200 s^−1^) were applied. The time interval between measurements was 10 s, and the duration of each measurement was 20 s. All tests were performed at room temperature (22 ± 2 °C).

*GC–MS Analysis*

The composition of the EOs was determined using a Polaris Q MS fitted with a Focus GC and a Triplus autosampler (Thermo Fisher Scientific, Waltham, MA, USA), as well as a split/splitless injector. The GC-MS system was equipped with a DB-5MS capillary column (25 m × 0.25 mm × 0.25 μm), and the analysis was carried out using helium as the carrier gas at a flow rate of 1.0 mL/min in constant flow mode. The temperature conditions were as follows: an initial temperature of 60 °C for three minutes, increased to 200 °C at a rate of 10 °C/min, held for two minutes, then increased at a rate of 12 °C/min to a final temperature of 240 °C. The injector temperature was held at 150 °C, the transfer line temperature at 250 °C, and the ion source temperature at 200 °C. The MS was operated in electron ionization mode (70 eV), scanning from 35 to 300 amu. The GC–MS was programmed to perform a 1.0 µL splitless injection. The separated components were identified using their Kovats retention indices (RI), computed against the n-alkanes (C8–C24) series (Sigma-Aldrich, St. Louis, MS, USA). Identification was validated by comparing data obtained by MS with the NIST mass spectral library and the literature. EO samples were diluted (1/100 *v*/*v*) in hexane prior to injection into the sampling port. The percentage composition of the identified compounds was calculated from the total ion chromatogram based on GC peak areas [79].

The volatile constituents of the formulations and honeys were collected using the Triplus autosampler’s headspace system. Approximately 3 g of sample was introduced into a 20 mL headspace vial, which was sealed with a silicone rubber septum and aluminum cap. The vial containing the samples was then heated to 80 °C for 10 min, after which 500 µL of headspace gas was injected into the column. The samples were then analyzed using the same temperature conditions as for GC–MS analysis.

#### 3.3.2. In Vivo Evaluation of Topical Structured Systems

The tests were performed using the Multi Probe Adapter MPA 6 system (Courage + Khazaka electronic GmbH, Cologne, Germany). Skin pH values were determined using a dedicated pH meter probe. The hydration level of the skin was assessed with the Corneometer^®^, while transepidermal water loss (TEWL) was evaluated using the Tewameter^®^. The melanin index and erythema level were measured with the Mexameter^®^. Skin elasticity was assessed using the Cutometer^®^, while the mechanical resistance and deformation behavior of the skin were analyzed with the Indentometer^®^. Skin microrelief and surface topography were analyzed using the Visioscan^®^ VC 20plus. All measurements were carried out under controlled temperature and humidity conditions, in accordance with the manufacturer’s recommendations.

*Study Design*

The testing was conducted on a total of 17 healthy volunteers aged between 24 and 87 years, of whom 10 were women, and 7 were men. Depending on skin type, 8 volunteers had combination skin, 5 had normal skin, and 4 had dry skin. According to skin phototype, 5 volunteers presented with phototype I, 6 with phototype II, and 6 with phototype III. Additionally, among the selected volunteers, 4 are smokers, and 13 are non-smokers.

Before enrolling in the study, all volunteers were examined by a dermatologist. Of the first 20 volunteers, only 17 received approval from the doctor.

All volunteers enrolled in the study were informed of the purpose of the research and provided informed consent before being included. Additionally, the volunteers were informed about each formulation individually. To protect confidentiality, each volunteer was assigned a numeric code from 1 to 17. The individuals included in the study did not show any sensitivity or active dermatological conditions. The tests were conducted over a period of 4 weeks under controlled laboratory conditions. Evaluations were carried out at six different time points: before the first application (initial time), 30 min after the first application, after 1 week, 2 weeks, 3 weeks, and 4 weeks of daily use. In the laboratory, the tests were conducted at a temperature of 22 ± 0.5 °C and a relative humidity of approximately 45%.

The formulations were applied to the forearm. The subareas were carefully delineated for each volunteer so that the analyzed zones were clearly separated. The new topical structured systems were used daily in the evening before bedtime. Volunteers were instructed not to apply any other cosmetic or dermatological products to the evaluated areas throughout the study period to avoid the occurrence of cross-contamination. The obtained results were expressed as mean ± standard error (SE).

The University of Medicine and Pharmacy of Craiova’s Commission for University and Scientific Ethics and Deontology approved the study (approval no. 72/11 May 2021), which was carried out in compliance with the Declaration of Helsinki.

## 4. Conclusions

The present study demonstrated the successful development of honey-based oil-in-water topical structured systems enriched with vegetable and essential oils intended for topical skin application. Physicochemical and rheological analyses showed that all formulations exhibited suitable semisolid characteristics, including pseudoplastic and thixotropic behavior, supporting their applicability as structured topical systems. The newly developed topical structured systems may contribute to skin barrier support, improvement of skin surface properties, and skin repair-associated effects.

The headspace GC-MS analysis confirmed the presence of characteristic terpene-rich volatile profiles associated with the incorporated essential oils, suggesting the retention of bioactive volatile compounds within the topical matrices. The identified constituents included compounds previously associated with antioxidant, soothing, antimicrobial, and anti-aging-related skin effects.

The in vivo evaluations performed over four weeks demonstrated good skin compatibility and beneficial effects on multiple skin biofunctional parameters, including hydration, elasticity, skin smoothness, wrinkle-related parameters, and surface homogeneity. The most pronounced moisturizing effect was observed in formulation F3, while formulation F1 showed notable improvements in parameters related to skin texture and wrinkle appearance.

Overall, the results suggest that honey- and essential oil-based structured systems are promising multifaceted semisolid formulations for skin care applications, combining favorable rheological behavior with beneficial effects on skin barrier function and appearance.

## Figures and Tables

**Table 1:** The Organoleptic Evaluation of Formulations.

Formulations	Color	Odor	Texture
F1	yellow	typical of the formulation components	firm, compact, slightly greasy.
F2	cream	typical of the formulation components	soft, smooth, less greasy.
F3	yellow	typical of the formulation components	moderate, slightly oily, balanced.

**Table 2:** The density, pH and humidity values of formulations.

Formulations	Density (g/cm^3^) *	pH (22 °C) *	Humidity (%)
F1	0.95 ± 0.07	5.50 ± 0.03	67.41
F2	0.96 ± 0.02	5.60 ± 0.02	66.22
F3	0.92 ± 0.02	5.30 ± 0.02	69.05

* Data are expressed as mean ± SD (*n* = 3).

**Table 3:** Major volatile compounds identified by headspace GC-MS in formulation F1 and their probable origin.

Compound	Relative Area (%)	Probable Source/Remarks
Benzyl alcohol	87.59 ± 6.89	Cosgard (added ingredient)/preservative
Geraniol	5.77 ± 0.61	Palmarosa essential oil/main marker of Palmarosa essential oil
α-Bisabolol	1.41 ± 0.09	Added ingredient
Linalool	1.23 ± 0.08	Palmarosa essential oil and Manuka honey
Geranyl acetate	1.08 ± 0.05	Palmarosa essential oil
Benzaldehyde	1.06 ± 0.05	Manuka honey/volatile aroma
Limonene	1.06 ± 0.05	Palmarosa essential oil
β-(E)-Caryophyllene	0.36 ± 0.01	Palmarosa essential oil
Myrcene	0.35 ± 0.01	Palmarosa essential oil
α-Citral (Geranial)	<0.10	Palmarosa essential oil/minor compound

**Table 4:** Major volatile compounds identified by headspace GC-MS in formulation F2 and their probable origin.

Compound	Relative Area (%)	Probable Source/Remarks
Benzyl alcohol	24.00 ± 2.44	Cosgard (added ingredient)/preservative
Camphene	39.36 ± 2.92	Cistus essential oil/major monoterpene
α-Pinene	21.45 ± 2.37	Cistus essential oil/major monoterpene
Tricyclene	8.04 ± 1.88	Cistus essential oil/characteristic terpene
Benzaldehyde	1.66 ± 0.11	Manuka honey-derived compound
Bornyl acetate	1.38 ± 0.09	Cistus essential oil/oxygenated monoterpene
p-Cymene	0.94 ± 0.05	Cistus essential oil/aromatic terpene
β-Pinene	0.89 ± 0.05	Cistus essential oil/monoterpene
Limonene	0.44 ± 0.02	Cistus essential oil/monoterpene
Myrcene	0.32 ± 0.02	Cistus essential oil/monoterpene
α-Terpinene	0.10 ± 0.00	Cistus essential oil/minor monoterpene
γ-Terpinene	<0.10	Cistus essential oil/minor monoterpene
α-Terpinolene	<0.10	Cistus essential oil/minor monoterpene
Linalool	<0.10	Cistus essential oil and Manuka honey/minor oxygenated monoterpene
Terpinen-4-ol	<0.10	Cistus essential oil/minor oxygenated monoterpene
Borneol	0.10 ± 0.00	Cistus essential oil/minor oxygenated monoterpene
α-Bisabolol	0.48 ± 0.04	Added ingredient

**Table 5:** Major volatile compounds identified by headspace GC-MS in formulation F3 and their probable origin.

Compound	Relative Area (%)	Probable Source/Remarks
Benzyl alcohol	58.91 ± 4.77	Cosgard (added ingredient)/preservative
cis-β-Ocimene	9.24 ± 0.98	Lavender essential oil/important lavender essential oil -related terpene
Linalool acetate	7.77 ± 0.82	Lavender essential oil/major lavender essential oil marker
Linalool	6.34 ± 0.64	Lavender essential oil/major lavender essential oil marker
trans-β-Ocimene	4.88 ± 0.42	Lavender essential oil/characteristic terpene
Benzaldehyde	3.36 ± 0.28	Possible honey-derived volatile
Myrcene	0.98 ± 0.08	Lavender essential oil/monoterpene
α-Pinene	0.87 ± 0.08	Lavender essential oil/minor monoterpene
Camphene	0.72 ± 0.07	Lavender essential oil/minor monoterpene
Terpinen-4-ol	0.60 ± 0.05	Lavender essential oil/oxygenated monoterpene
Lavandulyl acetate	0.56 ± 0.05	Lavender essential oil/characteristic lavender terpene
1-Octen-3-ol acetate	0.42 ± 0.03	Lavender essential oil/minor volatile ester
α-Thujene	0.35 ± 0.02	Lavender essential oil/minor terpene
β-(E)-Caryophyllene	0.32 ± 0.02	Lavender essential oil/sesquiterpene
β-Pinene	0.12 ± 0.01	Lavender essential oil/minor monoterpene
α-Terpinolene	0.15 ± 0.01	Lavender essential oil/minor monoterpene
Camphor	0.15 ± 0.01	Lavender essential oil/oxygenated monoterpene
Borneol	0.16 ± 0.01	Lavender essential oil/oxygenated monoterpene
α-Terpineol	0.11 ± 0.01	Lavender essential oil/minor oxygenated terpene
Allo-ocimene	0.11 ± 0.01	Lavender essential oil/minor monoterpene
α-Humulene	<0.10	Lavender essential oil/trace sesquiterpene
Lavandulol	<0.10	Lavender essential oil/trace lavender essential oil marker
α-Bisabolol	0.68 ± 0.05	Added ingredient

**Table 6:** Composition of the Experimental Formulations (% *w*/*w*).

Ingredients (% *w*/*w*)	F1	F2	F3
**Phase A—Aqueous Phase**			
Distilled water	62	62	62
Glycerin	4	4	4
Propanediol	2	2	2
Manuka honey	2.5	2.5	–
Tualang honey	2.5	–	2.5
Chestnut honey	–	2.5	–
Manna honey	–	–	2.5
**Phase B** (**Oil Phase**)			
Olivem 1000	6	6	6
Cetyl alcohol	3	3	3
Cholesterol	1	1	1
Deodorized cocoa butter	2	2	2
Aloe oil	7	5	–
Hemp seed oil	3	–	–
Pomegranate seed oil	–	5	–
Calendula oil	–	–	3
Centella oil	–	–	7
**Phase C** (**Cool-Down Phase < 40 °C**)			
Vitamin E	0.3	0.3	0.3
Low molecular weight hyaluronic acid	0.2	0.2	0.2
Niacinamide	2	2	2
Panthenol	1	1	1
Allantoin	0.2	0.2	0.2
Bisabolol	0.3	0.4	0.1
Coenzyme Q10	0.2	0.2	–
Ceramide NP	–	–	0.4
Palmarosa essential oil	0.2	–	–
Cistus essential oil	–	0.1	–
Lavender essential oil	–	–	0.2
Cosgard preservative	0.6	0.6	0.6

## Data Availability

The original contributions presented in this study are included in the article/Supplementary Material. Further inquiries can be directed to the corresponding authors.

## Supplementary Materials

The following supporting information can be downloaded at: https://www.mdpi.com/article/10.3390/ph19071103/s1; Table S1: Volatile compounds identified by headspace GC-MS in formulation F1 and corresponding raw materials; Table S2: Volatile compounds identified by headspace GC-MS in formulation F2 and corresponding raw materials; Table S3: Volatile compounds identified by headspace GC-MS in formulation F3 and corresponding raw materials; Figure S1. The organoleptic appearance of the developed formulations (F1, F2, F3); Figure S2. Packaging of the Formulations for Further Testing.
