# Supplementary material for: Development and Characterization of Honey- and Essential Oil-Based Structured Systems for Skin Applications
Source: Pharmaceuticals (Basel). 2026 Jul 17;19(7):1103. doi: 10.3390/ph19071103 (PMC13416500; doi:10.3390/ph19071103)
Supplement: Supplementary file 1 [file pharmaceuticals-19-01103-s001.zip › pharmaceuticals-4377879-supplementary.pdf]

Supplementary Materials

Table S1. Volatile compounds identified by headspace GC-MS in formulation F1 and corresponding raw materials.

Compound	RI	Relative Area (%)			
		F1	EO1	H1	H2
Isovaleraldehyde	657			8.21 ± 0.73	40.20 ± 3.98
Isopentyl alcohol	699			1.36 ± 0.08	6.37 ± 0.60
Furfural	799			8.12 ± 0.78	37.09 ± 3.53
Acetylfuran	915				1.38 ± 0.11
α-Pinene	934	<0.10			
Benzaldehyde	963	1.06 ± 0.05		3.33 ± 0.29	
5-Methylfurfural	967				2.32 ± 0.21
Myrcene	990	0.35 ± 0.01	0.15 ± 0.02		
Limonene	1027	1.06 ± 0.05	0.23 ± 0.02		
2-Methylbenzofuran	1028			9.53 ± 0.88	
cis-β-Ocimene	1033		0.41 ± 0.04		
Benzyl alcohol	1037	87.59 ± 6.89			
trans-β-Ocimene	1044		0.31 ± 0.02		
Benzeneacetaldehyde	1045			0.95 ± 0.08	0.77 ± 0.07
cis-Linalool oxide	1072			7.05 ± 0.66	0.48 ± 0.04
Linalool	1098	1.23 ± 0.08	1.99 ± 0.09	4.21 ± 0.39	
Isophorone	1125			2.47 ± 0.21	
Allo-Ocimene	1127		<0.10		
Lilac aldehyde A	1142			3.14 ± 0.29	
Ketoisophorone	1145			24.22 ± 2.12	
Lilac aldehyde B	1150			8.02 ± 0.78	
Lilac aldehyde D	1165			2.57 ± 0.21	
Myrtenol	1199			1.97 ± 0.18	
Safranal	1202			1.32 ± 0.09	
Verbenone	1225			0.91 ± 0.07	
Nerol (cis-geraniol)	1227		0.39 ± 0.03		
β-Citral (Neral)	1242		<0.10		
Geraniol	1251	5.77 ± 0.61	78.55 ± 6.12		
α-Citral (Geranial)	1269	<0.10	0.17 ± 0.01		
o-Acetanisole	1295			5.38 ± 0.51	
a-Cubebene	1349		<0.10		
Neryl acetate	1356		<0.10		
Geranyl acetate	1376	1.08 ± 0.05	13.15 ± 1.48		
β-(E)-Caryophyllene	1425	0.36 ± 0.01	3.34 ± 0.37		
Alloaromadendrene	1461		0.30 ± 0.01		
δ-Cadinene	1524		<0.10		
Butyl caprate	1594				9.41 ± 0.91
Caryophyllene oxide	1597		0.13 ± 0.01		
α-Bisabolol	1686	1.41 ± 0.09			
Total identified Compounds (%)		100.00 ± 7.84	99.39 ± 8.22	92.78 ± 8.41	98.04 ± 9.45

EO1: *Cymbopogon martinii* essential oil (palmarosa), H1: Manuka honey, H2: Tualang honey

Table S2. Volatile compounds identified by headspace GC-MS in formulation F2 and corresponding raw materials.

Compound	RI	Relative Area (%)			
		F2	EO2	H1	H4
Isovaleraldehyde	657			8.21 ± 0.73	
Isopentyl alcohol	699			1.36 ± 0.08	
Furfural	799			8.12 ± 0.78	
Tricyclene	922	8.04 ± 0.88	2.89 ± 0.23		
α-Pinene	934	21.45 ± 2.37	16.37 ± 1.59		
Camphene	949	39.36 ± 2.92	31.95 ± 3.02		
Benzaldehyde	963	1.66 ± 0.11		3.33 ± 0.29	
b-Pinene	977	0.89 ± 0.05	0.96 ± 0.08		
Myrcene	990	0.32 ± 0.02	0.30 ± 0.01		
α-Terpinene	1015	0.10 ± 0.00	<0.10		
p-Cymene	1023	0.94 ± 0.05	1.68 ± 0.11		
Limonene	1027	0.44 ± 0.02	0.39 ± 0.03		
2-Methylbenzofuran	1028			9.53 ± 0.88	
Benzyl alcohol	1037	24.00 ± 2.44			
2,2,6-Trimethylcyclohexanone	1038		2.67 ± 0.21		
Benzeneacetaldehyde	1045			0.95 ± 0.08	
γ-terpinene	1061	<0.10	0.66 ± 0.04		
cis-Linalool oxide	1072			7.05 ± 0.66	
a-Terpinolene	1090	<0.10	0.58 ± 0.04		
Linalool	1098	<0.10	0.16 ± 0.01	4.21 ± 0.39	
Isophorone	1125			2.47 ± 0.21	
α-Campholenal	1129	<0.10	0.25 ± 0.01		
Lilac aldehyde A	1142			3.14 ± 0.29	
trans-Pinocarveol	1144	<0.10	0.73 ± 0.05		
Ketoisophorone	1145			24.22 ± 2.12	
Lilac aldehyde B	1150			8.02 ± 0.78	
Camphor	1151	<0.10	0.76 ± 0.05		
Isoborneol	1163	<0.10	<0.10		
Lilac aldehyde D	1165			2.57 ± 0.21	
Borneol	1172	0.10 ± 0.00	2.26 ± 0.19		
Terpinen-4-ol	1180	<0.10	0.97 ± 0.08		
p-Cymen-8-ol	1187	<0.10	0.12 ± 0.00		
α-Terpineol	1193	<0.10	0.40 ± 0.02		
Myrtenol	1201	<0.10	0.32 ± 0.02	1.97 ± 0.18	
Safranal	1202			1.32 ± 0.09	
Verbenone	1225			0.91 ± 0.07	
Linalyl acetate	1253	<0.10	1.13 ± 0.09		
Bornyl acetate	1288	1.38 ± 0.09	18.13 ± 1.64		
o-Acetanisole	1295			5.38 ± 0.51	
Myrtenyl acetate	1326		<0.10		
a-Cubebene	1349	<0.10	0.65 ± 0.04		
Cyclosativene	1371	<0.10	1.44 ± 0.10		
α-Copaene	1377	<0.10	1.02 ± 0.08		
α-Gurjunene	1413		0.11 ± 0.00		
β-Caryophyllene	1425		0.31 ± 0.01		
γ-Muurolene	1478	<0.10	0.59 ± 0.04		
δ-Cadinene	1524	<0.10	3.58 ± 0.32		
a-Cadinene	1539		0.19 ± 0.00		

α-Calacorene	1551		0.23 ± 0.00		
Spathulenol	1590		0.23 ± 0.00		
Caryophyllene oxide	1597		0.19 ± 0.00		
Guaiol	1606		1.79 ± 0.12		
Di-epi-1,10-cubenol	1618		0.92 ± 0.05		
α-Cadinol	1650		0.13 ± 0.00		
α-Bisabolol	1686	0.48 ± 0.04			
**Total identified compounds (%)**		**99.76 ± 8.99**	**96.31 ± 8.29**	**92.78 ± 8.41**	**0**

EO2: *Cistus ladaniferus* essential oil, H1: Manuka honey, H4: Chestnut honey: no volatile compounds were detected by HS-GC-MS under the analytical conditions employed; therefore, the total identified volatile compounds were 0%.

Table S3. Volatile compounds identified by headspace GC-MS in formulation F3 and corresponding raw materials.

Compound	RI	Relative Area (%)			
		F3	EO3	H2	H3
Isovaleraldehyde	657			40.20 ± 3.98	8.06 ± 0.78
Isopentyl alcohol	699			6.37 ± 0.60	
Furfural	799			37.09 ± 3.53	56.85 ± 5.25
Acetylfuran	915			1.38 ± 0.11	1.04 ± 0.08
α-Thujene	926	0.35 ± 0.02	0.19 ± 0.01		
α-Pinene	934	0.87 ± 0.08	0.15 ± 0.01		
Camphene	949	0.72 ± 0.07	0.23 ± 0.01		
Benzaldehyde	963	3.36 ± 0.28			
5-Methylfurfural	967			2.32 ± 0.21	1.06 ± 0.08
β-Pinene	977	0.12 ± 0.01	<0.10		
Myrcene	990	0.98 ± 0.08	0.72 ± 0.05		
p-Cymene	1023	0.62 ± 0.05	0.47 ± 0.03		1.69 ± 0.12
cis-β-Ocimene	1033	9.24 ± 0.98	8.43 ± 0.79		
Benzyl alcohol	1037	58.91 ± 4.77			
trans-β-Ocimene	1044	4.88 ± 0.42	3.57 ± 0.31		
Benzeneacetaldehyde	1045			0.77 ± 0.07	
g-Terpinene	1061	0.19 ± 0.01	0.17 ± 0.01		
cis-Linalool oxide	1072			0.48 ± 0.04	2.57 ± 0.22
p-Cymenene	1087				16.22 ± 1.54
α-Terpinolene	1090	0.15 ± 0.01	0.23 ± 0.01		
Linalool	1098	6.34 ± 0.64	19.23 ± 1.85		
1-Octen-3-ol acetate	1105	0.42 ± 0.03	0.58 ± 0.04		
Allo-ocimene	1127	0.11 ± 0.01	0.48 ± 0.04		
cis-β-Terpineol	1144		<0.10		
Camphor	1151	0.15 ± 0.01	0.42 ± 0.03		
Lavandulol	1166	<0.10	0.52 ± 0.04		
Borneol	1172	0.16 ± 0.01	1.00 ± 0.08		
Terpinen-4-ol	1180	0.60 ± 0.05	3.34 ± 0.29		0.69 ± 0.05
p-Cymen-8-ol	1185	<0.10	0.33 ± 0.02		7.25 ± 0.69
α-Terpineol	1193	0.11 ± 0.01	1.23 ± 0.08		
Nerol (cis-geraniol)	1227		0.12 ± 0.00		
Isoborneol, formate	1232	<0.10	0.18 ± 0.00		
Benzaldehyde, 4-(1-methylethyl)-	1245		0.14 ± 0.00		
Linalool acetate	1253	7.77 ± 0.82	35.40 ± 3.29		
Dihydrolinalool acetate	1273		<0.10		

Lavandulyl acetate	1285	0.56 ± 0.05	4.02 ± 0.39		
α-Terpineol acetate	1348		<0.10		
Neryl acetate	1356		0.39 ± 0.03		
Geranyl acetate	1376		0.64 ± 0.05		
cis-α-Bergamotene	1412		<0.10		
β-(E)-Caryophyllene	1425	0.32 ± 0.02	6.76 ± 0.60		
trans-α-Bergamotene	1433		0.26 ± 0.01		
cis-β-Farnesene	1441		<0.10		
α-Humulene	1450	<0.10	6.00 ± 0.51		
trans-β-Farnesene	1454		0.18 ± 0.01		
Alloaromadendrene	1461		0.25 ± 0.01		
Germacrene D	1488		0.86 ± 0.05		
γ-Cadinene	1520		0.28 ± 0.01		
δ-Cadinene	1524		0.11 ± 0.00		
Butyl caprate	1594			9.41 ± 0.91	
Caryophyllene oxide	1597		0.38 ± 0.02		
α-Cadinol	1650		0.21 ± 0.01		
α-Bisabolol	1686	0.68 ± 0.05			
Total identified compounds (%)		97.81 ± 8.48	97.85 ± 8.72	98.04 ± 9.45	95.88 ± 8.73

EO3: *Lavandula angustifolia* essential oil, H2: Tualang honey, H3: Manna honey

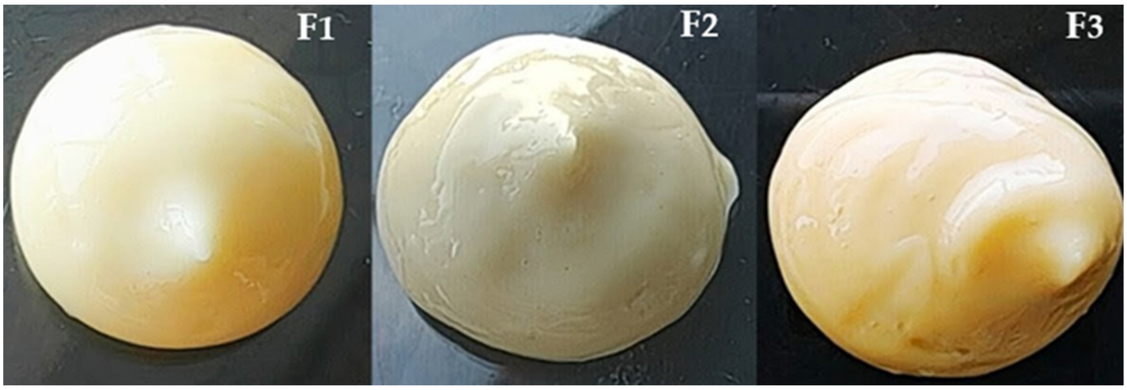

Figure S1. The organoleptic appearance of the developed formulations (F1, F2, F3).

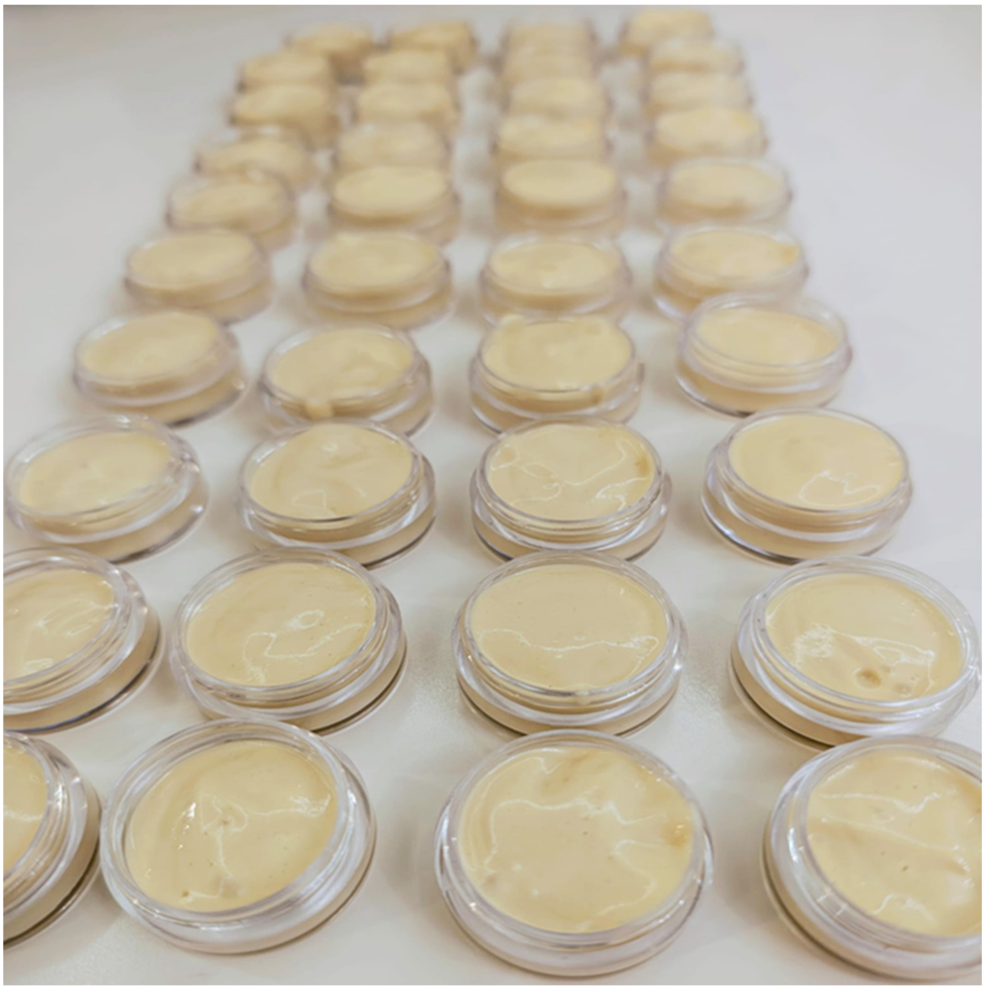

Figure S2. Packaging of the Formulations for Further Testing.
